# New York Academy of Medicine

**Published:** 1864-01

**Authors:** 


New York Academy of Medicine.—The annual oration before the Academy of Medicine was delivered on Thursday evening, December 10th, at the hall of the University College, by Prof. John W. Draper. The subject, was the Influence of History upon the Medical Profession, and it was treated by the distinguished orator with the most consummate ability. His studies of history enabled him to illustrate his subject with many exquisite sketches, and enrich it with many philosophical deductions. The audience was large and select, and received the address with great favor.—*Medical Times.*

